# Making Ends Meet: Microwave-Accelerated Synthesis of Cyclic and Disulfide Rich Proteins Via In Situ Thioesterification and Native Chemical Ligation

**DOI:** 10.1007/s10989-012-9331-y

**Published:** 2012-10-14

**Authors:** Sunithi Gunasekera, Teshome L. Aboye, Walid A. Madian, Hesham R. El-Seedi, Ulf Göransson

**Affiliations:** 1 Division of Pharmacognosy, Department of Medicinal Chemistry, Biomedical Centre, Uppsala University, Box 574, 751 23 Uppsala, Sweden; 2 Department of Chemistry, Faculty of Science, El-Menoufia University, 32512 Shebin El-Kom, Egypt

**Keywords:** Fmoc SPPS, Microwave assisted peptide synthesis, Native chemical ligation, Di-Fmoc-3,4-diaminobenzoic acid, Cyclotide, kalata B1, SFTI-1

## Abstract

The development of synthetic methodologies for cyclic peptides is driven by the discovery of cyclic peptide drug scaffolds such as the plant-derived cyclotides, sunflower trypsin inhibitor 1 (SFTI-1) and the development of cyclized conotoxins. Currently, the native chemical ligation reaction between an N-terminal cysteine and C-terminal thioester group remains the most robust method to obtain a head-to-tail cyclized peptide. Peptidyl thioesters are effectively generated by Boc SPPS. However, their generation is challenging using Fmoc SPPS because thioester linkers are not stable to repeated piperidine exposure during deprotection. Herein we describe a Fmoc-based protocol for synthesizing cyclic peptides adapted for microwave assisted solid phase peptide synthesis. The protocol relies on the linker Di-Fmoc-3,4-diaminobenzoic acid, and we demonstrate the use of Gly, Ser, Arg and Ile as C-terminal amino acids (using HBTU and HATU as coupling reagents). Following synthesis, an *N*-acylurea moiety is generated at the C-terminal of the peptide; the resin bound acylurea peptide is then deprotected and cleaved from the resin. The fully deprotected peptide undergoes thiolysis in aqueous buffer, generating the thioester in situ. Ultimately, the head-to-tail cyclized peptide is obtained via native chemical ligation. Two naturally occurring cyclic peptides, the prototypical Möbius cyclotide kalata B1 and SFTI-1 were synthesized efficiently, avoiding potential branching at the diamino linker, using the optimized protocol. In addition, we demonstrate the possibility to use the approach for the synthesis of long and synthetically challenging linear sequences, by the ligation of two truncated fragments of a 50-residue long plant defensin.

**Electronic supplementary material:**

The online version of this article (doi:10.1007/s10989-012-9331-y) contains supplementary material, which is available to authorized users.

## Introduction

The discovery of proteins whose ends are linked together to produce a circular topology is an unique event in protein biochemistry. Even though naturally occurring cyclic peptide products, such as cyclosporine, have been known for years, the presence of genetically encoded circular proteins in nature became known only recently. Today it has been established that organisms as diverse as bacteria, plants, fungi and mammals contain the genomic blueprints that are directly translated by nature’s ribosomal machinery into circular proteins (Göransson et al. 2012). Currently, the cyclotides (Craik et al. 1999) form the largest family of circular proteins with more than 200 characterized members. These proteins originate from plants and are commonly found within the plant families Rubiaceae and Violaceae.

Cyclotides have captured significant interest because they possess an ultra stable scaffold, the cyclic cystine knot (CCK) motif, derived from the circular backbone and three knotted disulfide bonds. In nature, cyclotides are presumably produced by plants to protect themselves against insects (Jennings et al. 2001), although other bioactivities, including anti-tumor (Lindholm et al. 2002; Svangård et al. 2004), anti-bacterial (Gran et al. 2008; Pränting et al. 2010), uterotonic (Gran et al. 2008) and anti-HIV (Gustafson et al. 2004), have been reported. A number of 50,000 different cyclotide variants are predicted to be present within the plant family Rubiaceae alone; thus cyclotides represent a natural combinatorial template having their inter-cysteine regions occupied by hypervariable sequences (Wang et al. 2008). Cyclotides are currently a target for bioengineering to take advantage of their plasticity and exceptional stability: bioactive epitopes pertaining to angiogenesis (Chan et al. 2011), anti-angiogenesis (Gunasekera et al. 2008) and protease inhibitory activities (Thongyoo et al. 2008; Thongyoo et al. 2009) have been successfully grafted onto the cyclotide framework.

**Fig. 1:**
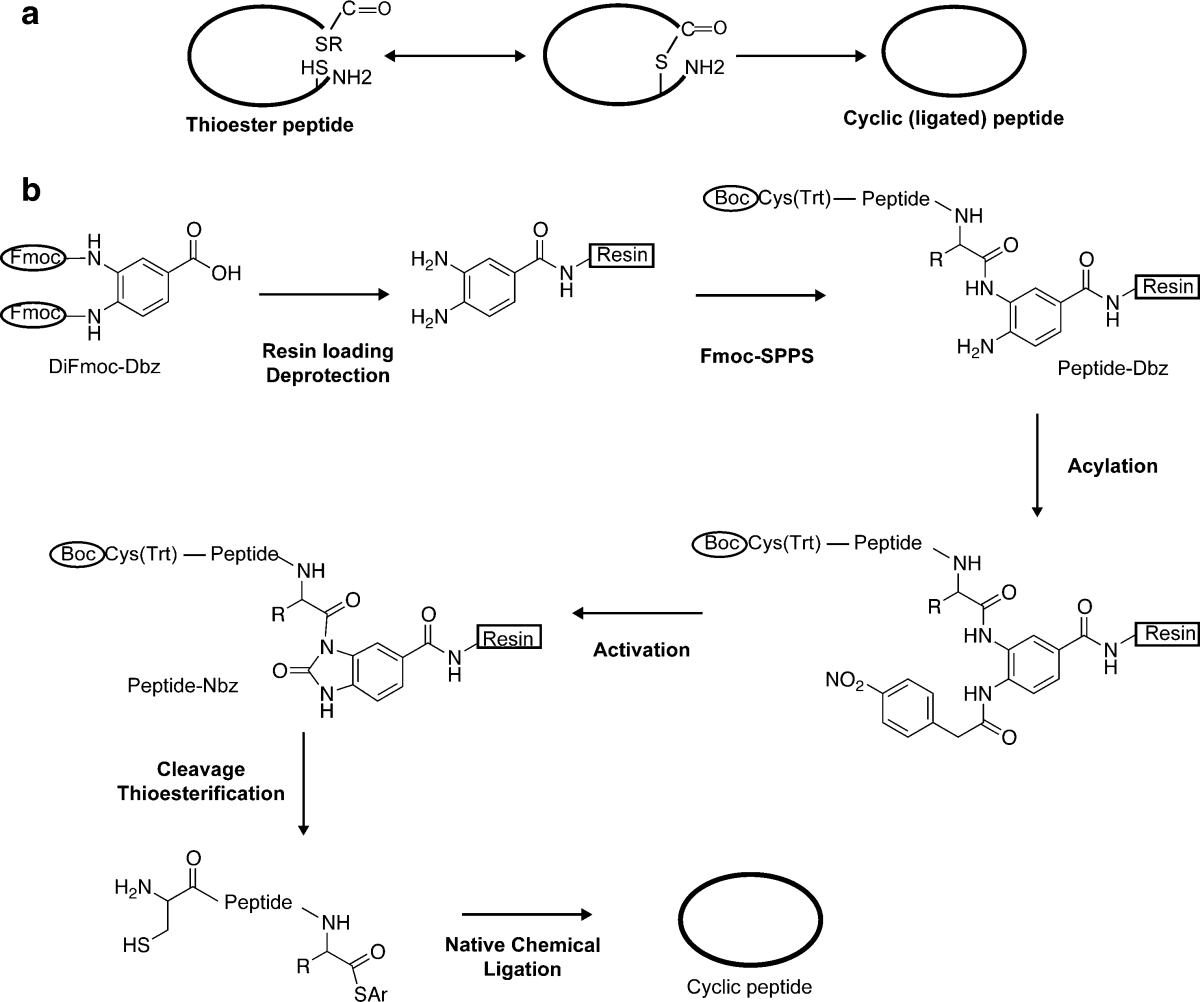
Schematic illustration of Native Chemical Ligation (NCL) and *N*-acylurea based Fmoc SPPS synthesis of cyclic peptide synthesis. **a** The thiolate group of the N-terminal cysteine residue attacks the C-terminal thioester in the unprotected peptide in an aqueous buffer at pH 7.0. This reversible transthioesterification step is chemo- and region-selective and leads to the formation of a thioester intermediate. That intermediate rearranges by an intramolecular S,*N*-acyl shift to form the head-to-tail cyclized peptide. **b** In the current work, Di-Fmoc-3,4-diaminobenzoic acid is used as a fully protected linker for the first coupling to the rink amide resin. The linker is then deprotected and peptide chain is elongated along one of the free amino groups. Following coupling the N-terminal Boc-Cys, the free amino group on the linker is acylated and thus activated, to yield the resin bound *N*-acyl urea peptide. *N*-acylurea peptide is then fully deprotected and cleaved from resin by strong cleavage. In situ thioesterification and cyclization according to **a** then leads to peptide ligation via an amide bond. A topologically circular molecule can be obtained by NCL when the N-terminal cysteine and the C-terminal thioester group are incorporated in the same peptide. Alternatively, the truncated counterpart synthetic peptide fragments can be ligated together to derive such long and difficult to synthesize peptides

Another example of a ribosomally synthesized plant-derived cyclic peptide is the 14-residue long sunflower trypsin inhibitory 1 (SFTI-1) that was originally discovered from the seeds of *Helianthus annus* (Luckett et al. 1999). Within its compact structure, SFTI-1 combines a single disulfide bond, a head-to-tail cyclized backbone and a network of internal hydrogen bonds (Korsinczky et al. 2001). Native SFTI-1 has subnanomolar trypsin inhibitory activity, and synthetic analogues of SFTI-1 have been developed into inhibitors of proteases pivotal to the progression of breast and prostate cancers (Long et al. 2001; Boy et al. 2010). Recently, proangiogenic epitopes were successfully grafted onto SFTI-1 (Chan et al. 2011). Lack of complete understanding of biosynthesis and peptide cyclization mechanisms for cyclotides and SFTI-1 has restricted their production by genetic approaches, and currently chemical synthesis remains the most robust approach to produce these molecules. With the rising interest in cyclotides and SFTI-1 as peptide stabilizing templates, it is becoming timely to explore novel approaches for their syntheses. Furthermore, the capacity to chemically synthesize these cyclic peptide templates also provides opportunities to explore the structure–function relationships of individual amino acids via synthetic mutants. Methods of peptide backbone cyclization also have general applicability, with several lines of evidence indicating that cyclization is advantageous in terms of augmenting the stability and bioavailability of therapeutically active peptides such as conotoxins (Clark et al. 2010; Lovelace et al. 2011). The native chemical ligation (NCL) reaction (Dawson et al. 1994) is one of the most popular strategies to join two unprotected peptide segments to each other through a native peptide bond, and can be used for both the synthesis of circular proteins and peptides, and for segmented synthesis of long and difficult peptide sequences (Hackeng et al. 1999; Clark and Craik 2010; Aboye et al. 2011). The ligation requires an N-terminal Cys and a C-terminal α-thioester, as outlined in Fig. 1a.

Unprotected C-terminal peptide thioesters, which are the key starting material for NCL, can be directly assembled on resin by Boc SPPS because the thioester is stable to the acidic deprotection treatment used in Boc SPPS. However, Boc SPPS has some drawbacks that make it unsuitable for many laboratories: hazardous HF is used for the final cleavage of the thioester peptide from the resin and the use of the strong acid is incompatible with many posttranslational modifications (Mende and Seitz 2011). On the other hand, thioester peptides are not directly accessible by Fmoc SPPS owing to the nucleophilicity of the Fmoc-deprotecting agent piperidine, although the Fmoc SPPS approach may be considered more user friendly. Several methods have been developed to overcome the problem of thioester instability in Fmoc SPPS; the use of special linkers such as Kenner’s safety catch linkers, using non-nucleophilic Fmoc deprotection cocktails or generating/activating the thioester group subsequent to the peptide assembly (Kenner et al. 1971; Clippingdale et al. 2000; Thongyoo et al. 2006; Park et al. 2010).

Recently, Dawson and coworkers described a method for generation of peptide thioesters compatible with Fmoc SPPS, based on a C-terminal *N*-acylurea peptide moiety (Blanco-Canosa and Dawson 2008). As illustrated in Fig. 1b, *O*-aminoanilides are used as stable synthetic intermediates in that method, which in turn are transformed to generate an *N*-acylbenzimidazolinone (*N*-acylurea) functionality following peptide assembly. Acidic deprotection gives acylurea peptide, which in turn undergoes thioesterification in neutral aqueous buffer. The C-terminal thioester peptide can then participate in NCL in the presence of the counterpart N-terminal peptide containing the Cys to join the N- and C-termini.

In the current work, we demonstrate the synthesis of disulfide-rich cyclic peptides with accelerated speed of synthesis and with high yields and purity using microwave assisted Fmoc SPPS. In addition, we demonstrate the use of the approach in a fragment-based synthesis of a larger defensin. The doubly protected Di-Fmoc-3,4-diaminobenzoic acid (illustrated in Fig. 1b) proves to be a useful linker, and Gly, Ser, Arg and Ile were successfully used as starting points for synthesis and subsequent conversion into thioesters.

## Materials and Methods

### Materials

All single Fmoc protected amino acids and Boc-Cys(Trt)-OH were from Iris Biotech (Marktredwitz, Germany) or PepChem (Tübingen, Germany). Tentagel R Ram Rink-type resin (0.18 mmol/g) and CLEAR amide resin (0.4 mmol/g) were from Peptide International (Louisville, KY). Fmoc-Pro NovaSyn TGT (0.2 mmol/g) and Fmoc-Asp(OtBu)-(Dmb)Gly–OH were from Novabiochem/Merck (Darmstadt, Germany). Di-Fmoc-3,4-diaminobenzoic acid was purchased from Anaspec (Fremont, CA). *O*-(7-Azabenzotriazol-1-yl)-*N,N,N′,N′*-tetramethyluronium hexafluorophosphate (HATU), *O*-(Benzotriazol-1-yl)-*N,N,N′,N′*-tetramethyluronium hexafluorophosphate (HBTU) and Fmoc protected amino acids were from PepChem. *N*,*N*-Dimethylformamide (DMF) and dichloromethane (DCM) were from Thermo Fisher Scientific (Waltham, MA). HPLC-grade acetonitrile (AcN) and formic acid (FA) were from VWR (West Chester, PA). Diethylether, diisopropylethylamine (DIPEA), guanidine hydrochloride, reduced and oxidized glutathione, piperidine, trifluoroacetic acid (TFA), triisopropylsilane (TIPS), 3,6-dioxa-1,8-octanedithiol (DODT), tris(2-carboxyethyl)phosphine hydrochloride (TCEP), 4-mercaptophenylacetic acid (MPAA) and 4-nitrophenyl chloroformate were from Sigma-Aldrich (St Louis, MO). PD-10 Sephadex G-25M columns were from GE Healthcare (Uppsala, Sweden).

### HPLC and MS

RP-HPLC was performed on an Äkta Basic (GE Healthcare) with detection at 215, 254 and 280 nm. Preparative HPLC was done on a 250 × 10 (i.d.) mm Phenomenex (Torrance, CA) C18 column (5 μm) at a flow rate of 4 ml/min, and analytical HPLC were done on a 150 × 2 (i.d.) mm Phenomenex C18 column (3 μm) at a flow rate of 0.3 μl/min. Solvents A (10 %AcN, 0.05 %TFA in water) and B (60 %AcN, 0.05 %TFA in water) were used in a linear gradient from of 0–70 % solvent B over 70 min for the preparative HPLC. A linear gradient from 0 to 100 % B over 30 min was used for analytical HPLC. MS and LC–MS were done on a LCQ classic (Finnigan/Thermo Electron, San Jose, CA) and a Q-Tof Micro coupled online with a nanoAcquity UPLC (Waters, Milford, MA), respectively. LC–UV–MS was done using an Acquity TUV detector (operated at 215 nm) coupled between the UPLC and the Q-Tof Micro. LC–MS was done at a flow rate of 0.3 μl/min on a Acquity UPLC BEH C18 column (1.7 μm, 150 × 0.075 mm) using solvents C (0.1 %FA in water) and D (0.1 %FA in AcN). A linear gradient from 0 to 60 % solvent D in solvent C over 40 min was used analysis. LC–UV–MS was done using a Phenomenex C18 column (150 × 1 mm, 5 μm) eluted at a flow rate of 40 μl/min and a linear gradient from 0 to 60 % solvent D in solvent C over 40 min was used for the analysis.

### Peptide Assembly

Kalata B1, SFTI-1_Arg_ and SFT1-1_Ile_ were synthesized on a Tentagel R Ram Rink-type resin (0.19 mmol/g) on 0.1 mmol scale. Defensin fragment 1 was synthesized on a CLEAR-amide resin using 0.25 mmol scale. Defensin fragment 2 was synthesized on 0.1 mmol scale using Fmoc-Pro-Novasyn TGT preloaded resin. The conditions used in Table 1 were used for the coupling of the Dbz linker and the amino acids to the corresponding resins. Coupling of the Dbz and the first C-terminal amino acid in kalata B1, SFTI-1_Arg_, SFTI-1_Ile_ and defensin peptide 1 were carried out manually. A microwave assisted Fmoc/HBTU SPPS protocol on a Liberty1 microwave peptide synthesizer (CEM Corp., Matthews, NC) was then used for elongation of the full sequences. Defensin fragment 2 was completely assembled in the automated synthesizer. Fmoc-amino acids were Fmoc-Ala-OH, Fmoc-Arg(Pbf)-OH, Fmoc-Asn(Trt)-OH, Fmoc-Asp(OtBu)-OH, Fmoc-Cys(Trt)-OH, Fmoc-Cys(Acm), Fmoc-Gln(Trt)-OH, Fmoc-Glu(OtBu)-OH, Fmoc-Gly-OH, Fmoc-Ile-OH, Fmoc-Leu-OH, Fmoc-Lys(Boc)-OH, Fmoc-Pro-OH, Fmoc-Ser(tBu)-OH, Fmoc-Thr(tBu)-OH, Fmoc-Trp(Boc)-OH, Fmoc-Tyr(tBu)-OH, Fmoc-Val-OH and the dipeptide Fmoc-Asp(OtBu)-(Dmb)Gly-OH. To protect the N-terminus of the peptide during subsequent acylation and to allow simultaneous deprotection, Boc-Cys(Trt)-OH was used in the final coupling step for kalata B1 and SFTI. For defensin fragment 1, a Boc-Arg was coupled at the N-terminal. The following conditions were used in the deprotection and coupling steps in the automated peptide synthesizer (outlined in detail in Table 1). Deprotection was repeated two times: 1 min (38 W, 70 °C) with 20 % piperidine in DMF (7 ml) followed by of 20 % piperidine in DMF (7 ml) for 3 min (63 W, 70 °C). The resin was washed with DMF (4 × 7 ml). Then 2.5 ml of the reagent mixture, containing amino acid (5 equiv.) HBTU (5 equiv.) and DIPEA (10 equiv.) were transferred to the reaction vessel. All amino acids were coupled for 5 min (32 W, 70 °C), except Cys and Arg. Cys was coupled for a total time of 6 min (2 min at room temperature, RT, followed by 4 min at 50 °C) and Arg was double coupled for 30 min (25 min at RT followed by 5 min of 75 °C). After completed synthesis, resins were washed (3 × 7 ml, DMF; 4 × 10 ml DCM) and dried under N_2_.

**Table 1:** Synthetic conditions used during peptide elongation

Step	Reagents	Target temperature^a^ and duration
Deprotection of resin	20 % piperidine, 7 ml	RT, 20 min × 1
Coupling of Dbz	Dbz (1 eqiv.), HBTU (2 eqiv.) DIPEA (3 eqiv.)	RT, 30 min × 2
Deprotection of Dbz	20 % piperidine, 7 ml	RT, 20 min × 2
Initial deprotection of aa	20 % piperidine, 7 ml	70 °C, 1 min
Deprotection of aa	20 % piperidine, 7 ml	70 °C, 3 min
Coupling of first aa		
Gly	aa (2 eqiv.), HBTU (2 eqiv.), DIPEA (3 eqiv.)	RT, 30 min
Ser	aa (4 eqiv.), HBTU (4 eqiv.), DIPEA (6 eqiv.)	RT, 30 min (× 2)
Ile/Arg	aa (6 eqiv.), HATU (6 eqiv.), DIPEA (9 eqiv.)	RT, 1 h × 2
Coupling of other aa		
aa other than Cys or Arg	aa (5 eqiv.), HBTU (5 eqiv.), DIPEA (10 eqiv.)	70 °C, 5 min
Cys	aa (5 eqiv.), HBTU (5 eqiv.), DIPEA (10 eqiv.)	RT, 2 min and 50 °C, 5 min
Arg	aa (5 eqiv.), HBTU (5 eqiv.), DIPEA (10 eqiv.)	RT, 25 min and 70 °C, 5 min

*aa* amino acid

^a^RT indicates coupling at room temperature; δT = 5 min under microwave heating

### Cleavage from Resin

Following synthesis, a sample of the resin (50 mg) was taken out and cleaved with TFA/TIPS/water (95.5:0.25:0.25, 2–3 h, RT). The cleaved peptide was filtered from resin, dried down to 0.5 ml with N_2_ and precipitated by the addition of cold diethylether. The precipitate was collected by centrifugation, redissolved in 50 % AcN/0.05 %TFA and freeze dried.

### Conversion of Dbz-Peptides into Nbz-Peptides

The resin bound peptide containing the Dbz linker was acylated using 4-nitrophenylchloroformate in DCM (16 equiv., 0.05 M, 55 min, RT). The resin was washed well with DCM and activated with 0.5 M DIPEA in DMF (195 equiv., 0.5 M, 20 min, RT). Resin was washed with DMF followed by DCM and dried under N_2_. The peptide-Nbz was cleaved from the resin using TFA/TIPS/water (95.5:0.25:0.25, 2–3 h). The precipitated peptide-Nbz was redissolved in 50 %AcN/0.05 %TFA in water and freeze dried.

### Cyclization/Ligation

A ‘one pot’ buffer for peptide cyclization/ligation was prepared using 200 mM MPAA, 20 mM TCEP and 6 M guanidine in a 200 mM phosphate buffer. The pH was adjusted to be between pH 7.0–7.2. Kalata-Nbz (1 mM) and SFTI-1 (2 mM) were incubated in the ‘one pot’ buffer for 24 h. For the ligation of the two defensin fragments, fragment 1-Nbz (2 mM) and fragment 2 (2.5 mM) were incubated together in the ‘one pot’ buffer for 24 h. After 24 h the ‘one pot’ buffer containing the cyclized products was desalted through a Sephadex PD 10 column. The column was equilibrated with 20 ml of 30 %AcN in water. The sample of cyclized peptide was dissolved and applied in 2 ml 30 %AcN solution and eluted with 30 %AcN in water (3 ml × 3 fractions). The fractions having the expected mass for the cyclic/ligated peptides were identified by MS and freeze dried.

### Oxidative Folding

Before oxidative folding, the cyclized kalata B1 was completely reduced using 10 mM DTT in 0.1 M NH_4_HCO_3_ buffer (pH 8.5) for 1 h, purified by RP-HPLC and freeze dried. Reduced, cyclized kalata B1 was then oxidized in a folding buffer of 0.1 M NH_4_HCO_4_ (pH 8.5) and isopropanol (50:50 v/v) containing 2 mM reduced glutathione and 0.4 mM oxidized glutathione for 24 h. After 24 h the reaction mixture was quenched with 0.4 %TFA and purified by RP-HPLC. SFTI-1 oxidized in the ‘one pot’ buffer for peptide cyclization/folding.

## Results

### Synthesis of the Cyclic Cystine Knotted kalata B1

The synthesis of the cyclotide kalata B1 was initiated on the Tentagel Rink amide resin with a C-terminal Di-Fmoc-3,4-diaminobenzoic acid (Di-Fmoc-Dbz) linker and an N-terminal Boc-Cys. Coupling the Di-Fmoc-Dbz linker to the resin and removal of its Fmoc protecting groups were carried out manually (outside the synthesizer) at RT. The choice of the Di-Fmoc-Dbz linker is slightly different from the Fmoc-3,4-diaminobenzoic acid (Fmoc-Dbz) linker that was originally reported (Blanco-Canosa and Dawson 2008). The Fmoc-Dbz linker has only one of the two amino groups protected by Fmoc whereas the linker in the current study has both amino groups at positions 3 and 4 protected. Complete coupling of the Di-Fmoc-Dbz occured at only 2 equiv. relative to the resin in contrast to 8 equiv. of Fmoc-Dbz as reported earlier (Blanco-Canosa and Dawson 2008). It appears likely that any potential oligomerization of the Dbz is avoided during coupling as both amino groups are protected, leading to more efficient coupling.

**Fig. 2:**
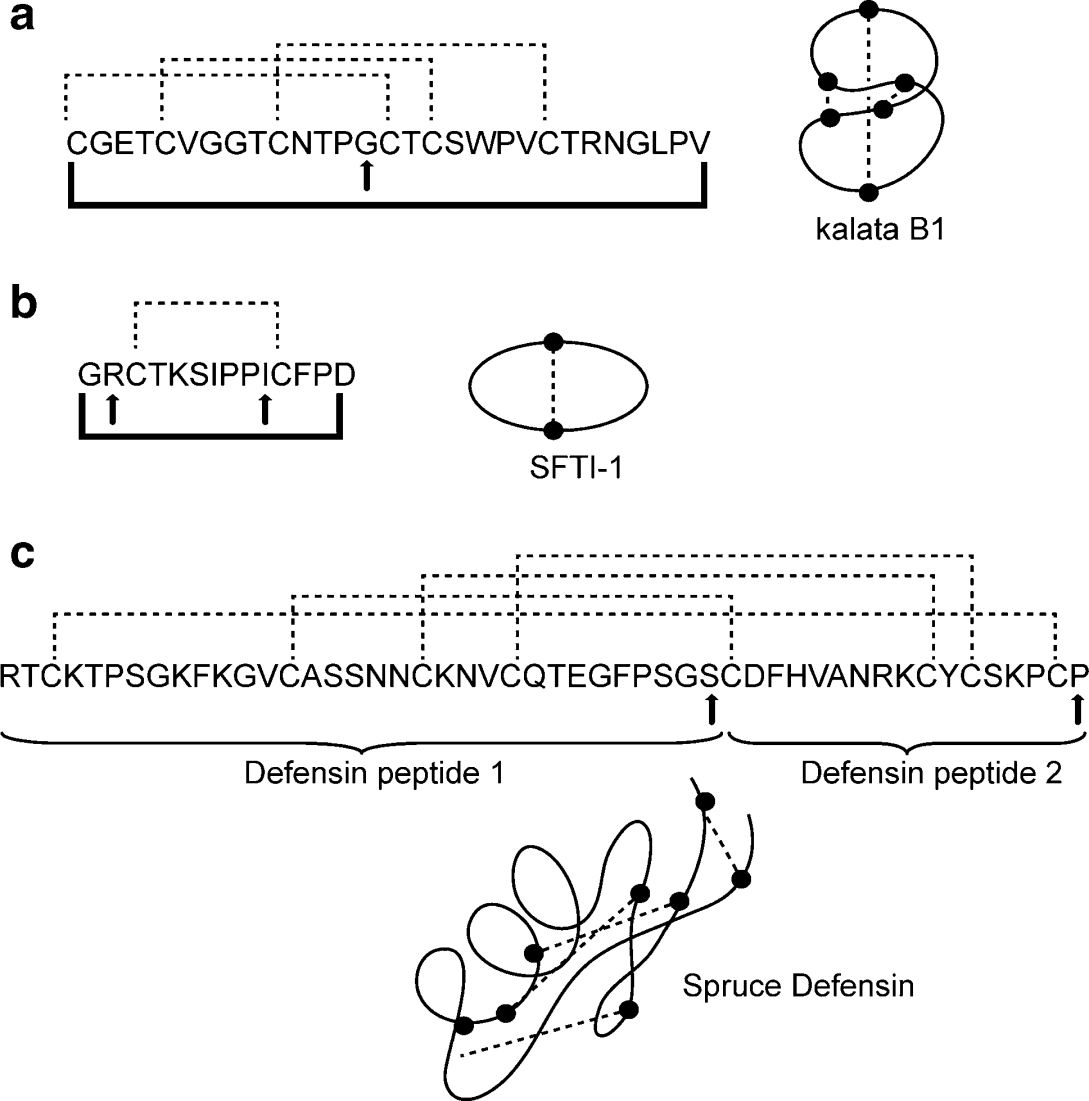
Sequences and schematic representation of the structures of peptides synthesized in the study. **a** kalata B1, **b** SFTI-1 and **c** the 50 amino acid long spruce defensin 1. The synthesis initiation point in each peptide is highlighted by *arrow*(*s*). *Dashed connecting lines* indicated the disulfide bonds and the bold connecting lines indicate the cyclized backbone

A complete summary of the synthesis conditions is given in Table 1. The Gly next to Cys (in loop 3) was selected as the initiation point for synthesis (highlighted by the arrow in Fig. 2) to prevent any possibility of epimerization and to facilitate fast reaction. In all there are five other possible synthesis initiation points next to the other Cys residues. In the method introduced by Blanco-Canosa and Dawson (2008) for the production of peptide thioesters, peptide chain extension ideally occurs only at one of the two unprotected amines of the Dbz-linker (either the amine in 3 or 4 position), whereas the other amine gets inactivated following initial acylation due to electronic and steric effects. However, one problem that have been observed for Gly-rich sequences and long, difficult to synthesize, peptides is the accumulation of acylated or branched products arising from the second, free, amine of Dbz (Mahto et al. 2011). To prevent branching, we coupled the first Gly at 1 equiv. relative to the resin as a single coupling (30 min, RT). The remaining portion of the peptide was then elongated in the automated peptide synthesizer by standard Fmoc SPPS with the assistance of microwave heating during deprotection and coupling steps. A crude yield of 63 % was obtained for kalata-Dbz by quantitative cleavage of a portion of the resin, and integration of the UV trace at 215 nm gave 76 % purity. Yields and purities are summarized in Table 2 and masses of synthetic products are shown in Table 3. Given that this yield was obtained after single couplings of all amino acids (except Arg) in the automated synthesizer using the default cycles from the manufacturer, and without monitoring coupling efficiency, there could be further room for improving the crude yield of the peptide. Expected amount of peptide-Dbz was calculated taking into account the amount of resin cleaved, increase in resin weight following synthesis, peptide molecular weight (deprotected and fully protected) and resin substitution value (see details in Supplementary Information, SI).

**Table 2:** Yield of kalata B1 and SFTI-1

Peptide	Peptide-Dbz recovery (%)^a^	Peptide-Dbz purity (%)^b^	Peptide-Nbz recovery (%)	Peptide-Nbz purity (%)	Native peptide yield (%)
Kalata B1	63	76	62	67	16
SFTI-1	81	79	89	88	50

^a^Peptide recovery is based on quantitative cleavage of peptide from resin

^b^Peptide purity is based on HPLC detection trace at 215 nm

**Table 3:** Expected and Observed peptide masses

Peptide	(M + H)^+^ calculated (observed)
Kalata-DBZ	3048.34 (3048.76)
Kalata-NBZ	3074.33 (3075.08)
Kalata B1 (cyclized and oxidized)	2891.19 (2891.86)
SFTI-DBZ	1666.88 (1665.94)
SFTI-NBZ	1692.88 (1691.08)
SFTI-1 (cyclized and oxidized)	1513.72 (1512.72)
Defensin peptide 1	1970.84 (1969.84)
Defensin peptide 2-DBZ	3840.01 (3839.01)
Defensin peptide 2-NBZ	3866.01 (3865.10)
Defensin peptide (ligated)	5658.71 (5657.84)

**Fig. 3:**
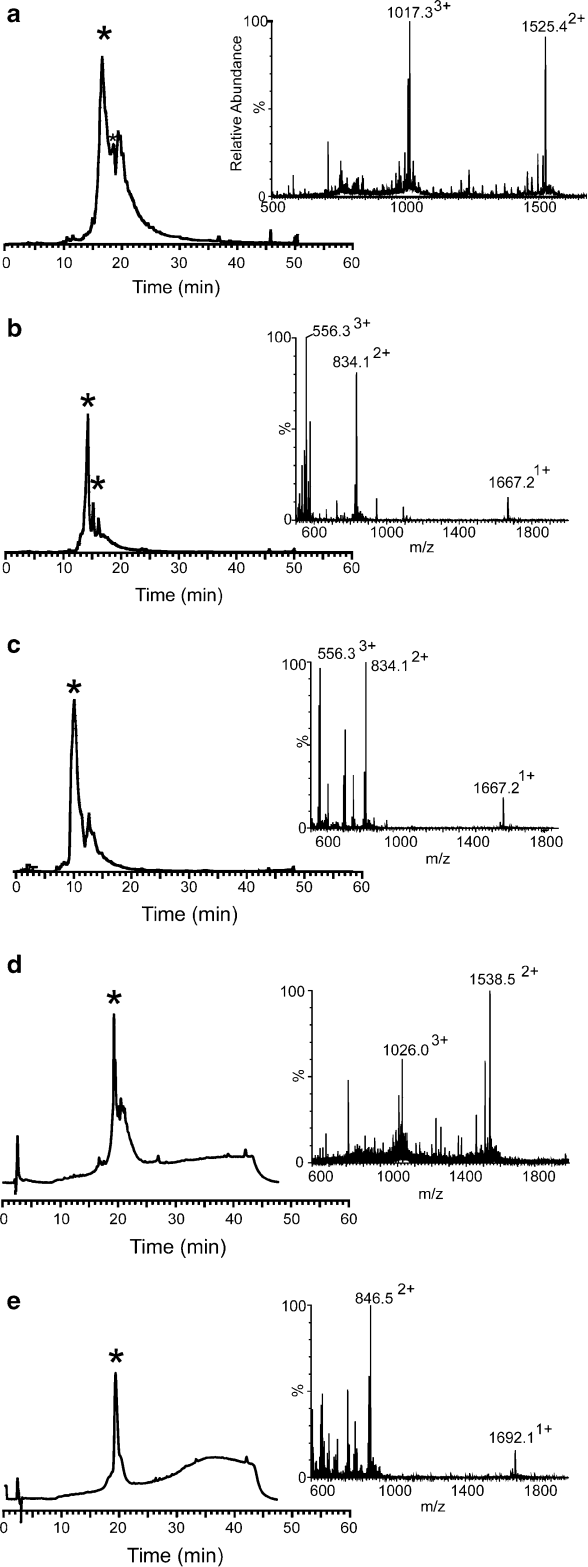
LC–UV–MS analysis of Dbz and Nbz peptides. **a** kalata B1-Dbz, **b** SFTI-1_Arg_-Dbz **c** SFTI-1_Ile_-Dbz **d** kalata-Nbz **e** SFTI-1_Arg_-Nbz. Chromatograms show the UV trace at 215 nm; mass spectra of the main peaks (marked by *) are shown to the *right*

As demonstrated in Fig. 3a, one of the isomeric forms of kalata-Dbz appeared as the main product on RP-HPLC analysis with the other isomeric form eluting very close to the main peak as a secondary peak. In addition, a by-product with a mass increase of 56.24 Da was observed. That mass increase may stem from a *tert*-butyl (tBu) protecting group (56.11 Da) or an extra Gly residue (57.05 Da). To rule out that the by-product was not due to the coupling of Gly on the free amino group on Dbz (i.e. from branching), MS–MS was done on the ion with the adduct and of the “native” ion. As shown in SI (Fig. 1), the analysis clearly showed that the unprotected amine on Dbz was not acylated, and we conclude that the extra mass was the result of a tBu-protecting group at a Cys or Thr in the sequence. Although we repeated our cleavage procedure by increasing the thiol scavenger (TFA/TIPS/DODT/water, 92.5.5/2.5/2.5/2.5), the peak with the extra mass of 56.24 was still observed as a minor peak.

Following synthesis, the peptide C-terminus was activated through acylation with *p*-nitrophenylchloroformate. Addition of base resulted in the resin-bound peptide benzimidazolinone (kalata B1-Nbz). After cleavage and deprotection, kalata B1-Nbz was obtained in 62 % yield and the purity was 67 % according to HPLC. No aminoanilide products were observed in the HPLC analysis, indicating that complete conversion of the benzimidazolinone has occurred. The crude peptide-Nbz was then incubated in the ‘one pot’ buffer for thioesterification and cyclization in situ. After 24 h, cyclized kalata B1 was purified using SEC with 80 % recovery.

**Fig. 4:**
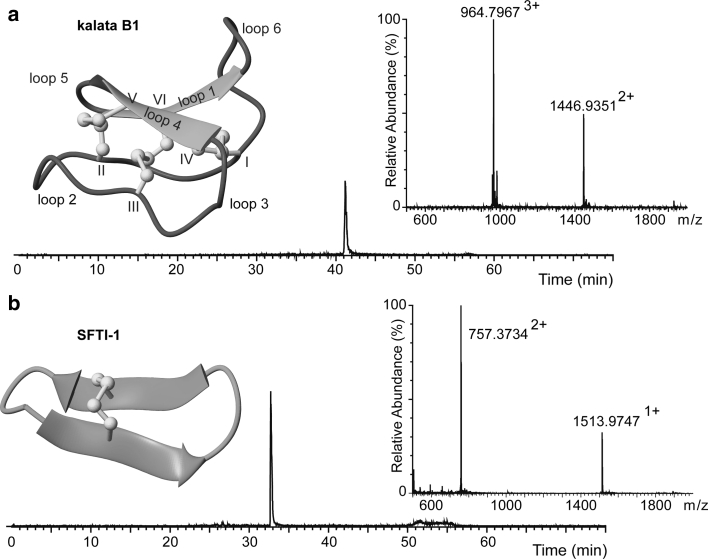
Analyses and structures of cyclic end products. Chromatograms show the co-injection of synthetic and native peptides on LC–MS; the mass spectra of the peaks, and the structures of **a** kalata B1 and **b** SFTI-1

In our early attempt to isolate cyclized kalata B1 by HPLC from the ‘one pot’ buffer, we observed that cyclized kalata B1 coeluted with thiols. Although extraction with diethylether enabled separation of the peptide from thiols, a significant amount of peptide (~50 %) could not be recovered through RP-HPLC. Cyclized kalata B1 collected by SEC contained oxidized Cys, but analytical RP-HPLC indicated several peaks with retention times earlier than the native kalata B1, indicative of misfolded kalata B1 (data not shown). Thus, cyclized kalata B1 was completely reduced and refolded in a buffer optimized for correct folding (Daly et al. 2003). Co-injection of 0.1 μl each of kalata B1 refolded (0.2 mg/ml) and native kalata B1 (0.2 mg/ml) confirmed the native conformation of synthetic kalata B1 (Fig. 4a). From 17.4 mg of peptide-Nbz, 4.2 mg of final pure kalata B1 was obtained. This corresponds to a total yield of 16 %, as calculated from the resin substitution value.

### Synthesis of the Cyclic SFTI-1

Coupling the Di-Fmoc-Dbz linker to the resin was carried out as previously described in the synthesis of kalata B1. Two SFTI-1 variants were made; initiated at Arg or at Ile as outlined in Fig. 2. In each case, the synthesis ended with an N-terminal Boc-protected Cys-residue. The coupling of the first amino acid in SFTI-1 variants (SFTI-1_Arg_ and SFTI-1_Ile_) gave incomplete coupling when HBTU was used as the coupling agent: despite increasing the amino acid and coupling agent equivalents or increasing the coupling incubation time as (see SFTI-1 syntheses a-d as described in SI). One challenge during peptide synthesis is *δ*-lactam formation when coupling Arg (Cezari and Juliano 1996). The formation of *δ*-lactam occurs when the internal nitrogen of the guanidine attacks the activated ester, irreversibly forming the lactam ring. This reaction is competitive with peptide-bond formation, and greatly reduces the coupling efficiency of Arg. It is possible that lactam formation is favored over the coupling of Arg to Dbz when HBTU is used as a coupling agent. Ile was also difficult to couple to the Dbz linker using HBTU. It is well established that *β*-branched amino acids are prone to form *β* sheets, often lead to aggregation during peptide strand elongation resulting in poor coupling efficiency (Garcia-Ramos et al. 2009). Thus, it appears that the structural features of the amino acid that is coupled to the Dbz linker could play an important role in the coupling efficiency. In contrast, the use of HATU as the coupling agent gave complete coupling of the first amino acids (Arg and Ile) to the Dbz linker, at 6 equiv. relative to the resin. The full sequence was then assembled using microwave assisted Fmoc SPPS on the Liberty1 synthesizer.

One of the most frequently encountered side reactions affecting Asp in Fmoc SPPS is aspartimide formation, resulting from a ring closure between the N of the α-carboxy amide bond and the *β*-carboxy side chain of the Asp with the loss of ester protecting group (Tam et al. 1988). The sequence Asp-Gly is prone to aspartimide formation, which in turn may undergo base catalysed epimerization to give α- and *β*-piperidide products in the presence of piperidine. SFTI-1 contains the problematic Asp-Gly sequence. In particular, when the synthesis of SFTI-1 is initiated at Arg, the Asp-Gly sequence is encountered early in the synthesis we anticipated that aspartimide formation is likely to occur due to repeated exposure to piperidine during synthesis. Thus, the Asp-Gly sequence was introduced in the form of Fmoc-Asp(OtBu)-(DMB)Gly-OH, with protecting groups on the N of α-carboxy amide and *β*-carboxy side chain, to circumvent the problem in the final syntheses. Analysis of crude SFTI-Dbz peptides by RP-HPLC revealed a prominent peak with the corresponding mass for SFTI-Dbz.

Similar to kalata-Dbz synthesis, a by-product with an extra mass of 56.24 Da was also found for SFTI-1-Dbz, as indicated in Fig. 3. However, in the case of SFTI, it is clear from the synthesis in which Asp-Gly was introduced as a dipeptide, that the adduct can not originate from branching at the second, free amine, of Dbz. (In that case, the adduct would have the mass of the dipeptide, not Gly alone). MS–MS analysis confirmed that there was no branching; again, a tBu group is the likely culprit for the by-product. The yield of the crude peptide-Dbz was 81 % for SFTI-1_Arg_-Dbz and 77 % for SFTI-1_Ile_-Dbz. HPLC integration peaks gave a purity of 79 and 80 %, respectively. Acylation, activation and cleavage of the acyl urea peptide were carried out for SFTI-1_Arg_-Dbz. SFTI-1_Arg_-Nbz was obtained in 89 % yield and the purity was 88 %. Yields and purities are summarized in Table 2 and masses of synthetic products are shown in Table 3.

After incubation of the crude *N*-acylurea peptide in the ‘one pot’ buffer for 24 h, cyclized SFTI-1 was purified by SEC with 80 % recovery. MS analysis demonstrated that cysteines were oxidized. Because SFTI-1 contains only one disulfide bond, it seemed likely that SFTI-1 attained its native conformation in the ‘one pot’ buffer. LC–MS analysis confirmed that that was the case: as shown in Fig. 4, co-injection of equal amounts of synthetic SFTI-1 and native SFTI-1 co-elutes as a single peak. On a preparative scale, synthetic SFTI-1 collected from SEC was purified by RP-HPLC as a final step. From 18.6 mg of peptide-Nbz, 10 mg of pure SFTI-1 was obtained. This corresponds to a total yield of 50 % of the final product (calculated from the resin substitution value).

### Synthesis of a Plant Defensin by Ligation of Fragments

Encouraged by the successful synthesis of kalata B1 and SFTI-1, we then decided to apply the same principles of *N*-acylurea mediated NCL in the synthesis of a 50-residue long plant defensin, spruce defensin (SPI1) (Sharma and Lonneborg 1996; Elfstrand et al. 2001). The defensin was made in two segments, a 33 residue long N-terminal peptide containing a C-terminal Dbz linker (Fragment 1) and the 17 residue C-terminal peptide (Fragment 2). In Fragment 1, the Di-Fmoc-Dbz group was coupled to a Clear amide resin and the synthesis was initiated at a Ser residue, and a Boc-Arg was introduced as the N-terminal residue. The peptide was elongated by Fmoc SPPS under microwave heating. Fragment 2 was entirely synthesized in the microwave synthesizer using a Pro preloaded resin. During the syntheses, small samples of the resins were cleaved to confirm synthesis accuracy. Fragment 2 (crude yield of 90 %) was subsequently purified by RP-HPLC.

**Fig. 5:**
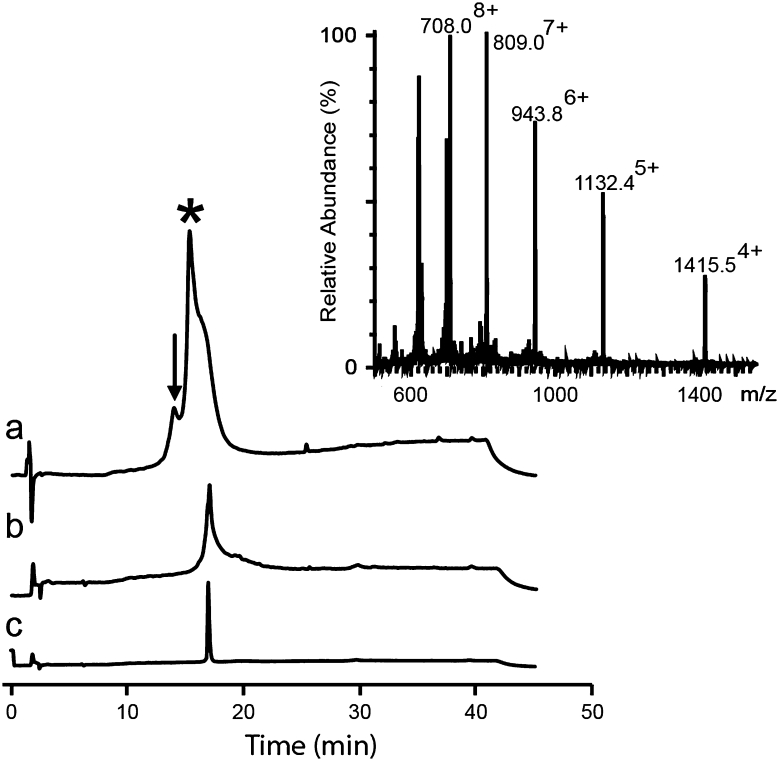
Analysis of truncated counterpart defensin peptides (fragment 1-Dbz and fragment 2) and the ligated spruce defensin peptide. **a** Ligated spruce defensin peptide is indicated by * with the corresponding MS trace. Hydrolyzed Nbz peptide is indicated by an *arrow*. **b** Defensin peptide 1-Dbz, and **c** defensin peptide 2

Fragment 1-Nbz was generated by the now established procedures for acylation, activation and cleavage. The two fragments were then incubated together in the ‘one pot’ buffer for 24 h using a slightly higher concentration of fragment 2 (2.5 mM for fragment 2 and 2 mM for fragment 1-Nbz). As indicated in Fig. 5, the ligation proceeded efficiently, and the only side product arose from trace hydrolysis. The ligated peptide was then collected by SEC and analysed by LC–MS; the ligated peptide appeared as a broad peak, presumably due to its larger size (5.6 kDa) and unfolded conformation.

## Discussion

In the current study we have used *N*-acylurea mediated native chemical ligation to generate two model cyclic peptides and a 50 residue long, linear peptide. The method relies on the synthesis of a peptide with a C-terminal Dbz linker and a N-terminal Cys, given the peptide is intended to be head-to-tail cyclized (kalata B1 and SFTI-1). Alternatively, a peptide synthesized with a C-terminal Dbz linker can be linked to a second peptide containing an N-terminal Cys, to yield one larger molecule (as in the case of the defensin). Following synthesis, the Dbz linker is activated to yield an *N*-acylurea moiety that acts as poor nucleophile to efficiently form a thioester at the C-terminal. In the presence of the N-terminal Cys, the thioester peptide then participates in NCL to form a native amide bond leading to either peptide cyclization or ligation.

There are several differences in our synthesis protocol from the one originally reported by Blanco-Canosa and Dawson (2008). The type of the Dbz linker used in the current study has both of its amino groups protected by Fmoc groups which is advantageous in terms of improving coupling efficiency as self-dimerization of the linker is prevented. In addition, we demonstrate that the strategy is compatible with microwave irradiation for deprotection and coupling to accelerate the speed of synthesis. Furthermore, we show that Arg, Ile and Ser can be used as starting points for syntheses; but it appears that the structural features of the amino acids play an important role in the coupling efficiency and requires case-specific optimization. Specifically, we found that Arg and Ile require HATU as a coupling agent, whereas Gly should be coupled at low equivalences to prevent possible branching of the second amino group on Dbz. Furthermore, due to the hydrophobicity of the peptides we have synthesized, their co-elution with thiols when separated by RP-HPLC has been problematic. Thus, in place of RP-HPLC, we have successfully used SEC to desalt the cyclized peptides.

SFTI-1 could be obtained in high total yields compared to kalata B1 (yields are summarized in Table 2). Because SFTI-1 is only 14 residues in length and also has only a single disulfide bond, its synthesis and folding are less complex than kalata B1. Following incubating SFTI-1-Nbz in the ‘one pot’ buffer, native SFTI-1 could be easily isolated by SEC and a single step of RP-HPLC. Kalata B1 synthesis is comparatively more laborious and results in lower yields than SFTI-1 because twice as many coupling cycles are needed to assemble the 29-residue peptide. Furthermore, two rounds of HPLC purification were required for kalata B1, first to isolate the fully reduced peptide and then to isolate the final native peptide following oxidative folding. However, it should be emphasized that no re-couplings (except for Arg) or optimizations were made for the automated peptide synthesis and the efficiency of each amino acid coupling was not monitored by the ninhydrin test. Most likely, there is room for further improvement of the current synthetic yields.

**Fig. 6:**
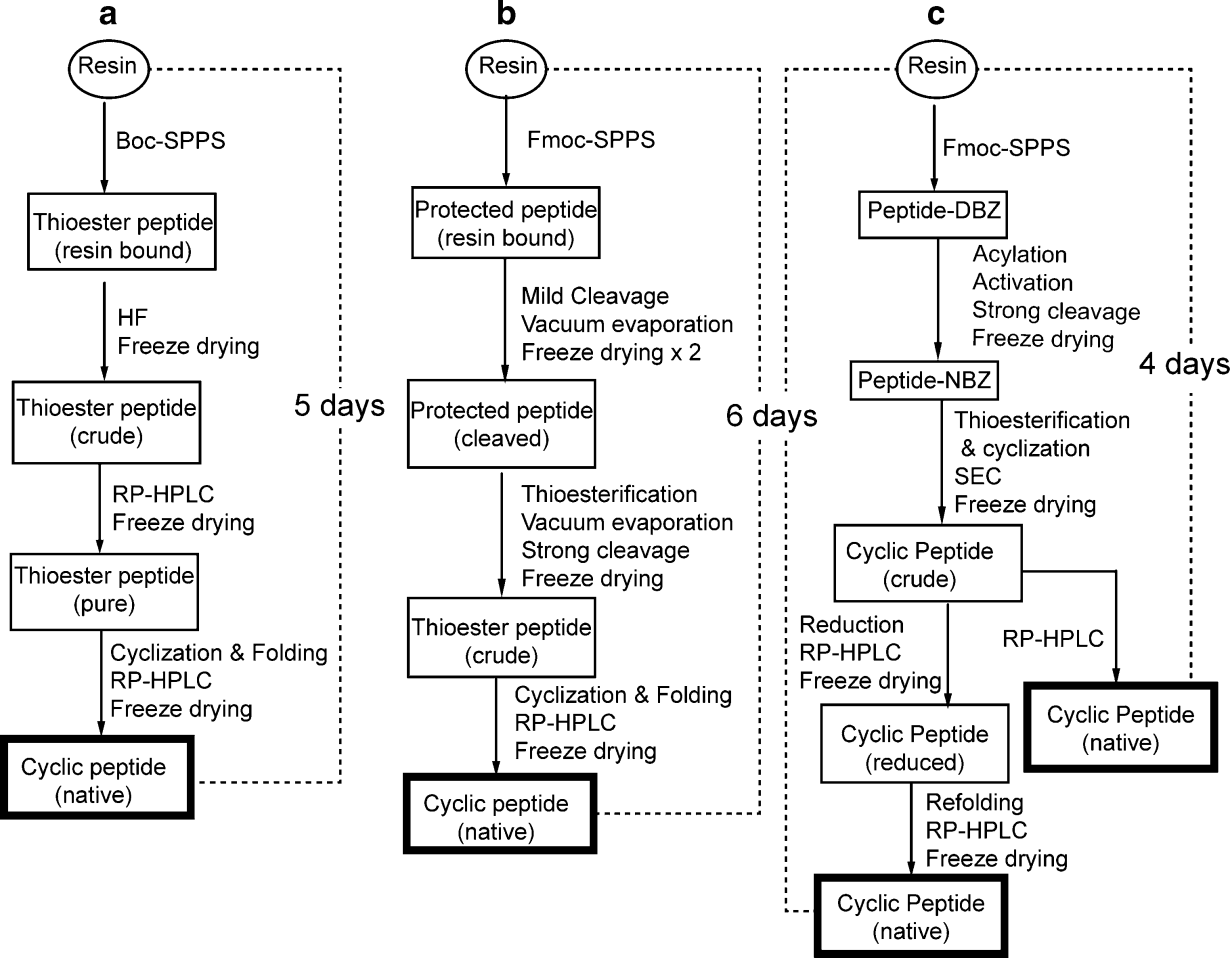
Different cyclotide synthesis strategies. **a** Boc SPPS strategy, **b** Fmoc SPPS strategy (based on thioesterification of protected peptide) and **c** the Fmoc SPPS strategy used in the current work (based on *N*-acylurea peptide intermediates)

Currently, there are several methods for the chemical synthesis of natural and engineered cyclotides, as outlined in Fig. 6. Boc SPPS based methods were the first to be developed for cyclotide synthesis and is still the dominating method. Boc SPPS is often referred to as the most robust method of cyclotide synthesis, simply because the linear cyclotide precursors can be conveniently generated on resin as crude thioester peptides. However, it should be noted that at least two rounds of purification are required, first to isolate the desired thioester peptides and then the cyclized and natively folded cyclotide, with each purification step often leading to some amount of unrecoverable peptide. The microwave assisted Boc SPPS strategy is reported to give >60 % yield of crude thioester peptide and 20 % yield of pure kalata B1 from thioester peptide.

The need to develop an Fmoc-based strategy without having to use HF in the final cleavage of peptide from resin and also with the opportunity to conveniently automate, drove the search of alternative denovo cyclotide synthesis methods. Thongyoo et al. successfully synthesized MCoTI-II by Fmoc synthesis based on a sulfonamide (‘safety catch’) linker, by activating the thioester precursors required for NCL following peptide elongation (Thongyoo et al. 2006). However, the resulting thioester peptide was isolated only in 15 % yield and the yield of native peptide is not reported. We have previously described a Fmoc SPPS protocol for cyclotide synthesis in which thioester peptide needed for NCL is generated in solution by reacting a protected peptide with thiols (Fig. 6b) (Leta Aboye et al. 2008; Park et al. 2010). This strategy gives good total yields for cyclotides, ~22 %kalata B1 and ~11 %MCoTI-I yields (yield calculated from the resins). One of the advantages of that strategy is that the crude thioester peptide can be directly converted into a natively folded, cyclized peptide in a ‘one pot’ buffer without an intermittent HPLC purification step. However, the need to make protected peptide has one drawback: the thioesterification step is extremely sensitive to any residual acid remaining from the mild acid cleavage, thus efficient vacuum evaporation (<2 mbar) of the protected peptide and at least two rounds of lyophilization are necessary to obtain protected peptide free of residual acid.

In comparison, the current protocol does not require a protected peptide to be cleaved off from the resin. The *N*-acylurea peptides can be directly generated and cleaved off from the resin followed by in situ thioesterification/cyclization in a ‘one pot’ buffer. Thus, high yield of cyclic/ligated peptide can be generated with accelerated speed. In case of SFTI-1, correct folding also takes place in the ‘one pot’ buffer, and the native peptide can be isolated in high yields. In comparison, kalata B1 was completely reduced and refolded to reach the native disulfide connectivity. The extra purification required contributes to the lower total yield of that peptide, but it should be noted that folding conditions have not yet been optimized: with the increasing knowledge of cyclotide oxidative folding (Aboye et al. 2011) it is likely that the extra purification step can be omitted in the future.

To date SFTI-1 has been chemically synthesized by different approaches. The most robust approach is via Boc SPPS in which the peptide is synthesized with C-terminal thioester linker and N-terminal Cys to facilitate cyclization by NCL (Daly et al. 2006). In addition, linear SFTI-1 precursors have been synthesized by Fmoc SPPS followed by head-to-tail cyclization to obtain the cyclic backbone with the assistance of coupling agents such as PyBop (Zablotna et al. 2002; Swedberg et al. 2011). In that case, an amino acid side chain protected peptide precursor is usually required to ensure that amide bond formation occurs only between an activated C-terminal carboxylate and an N-terminal Cys. Thus, two cleavage steps are needed: ‘mild’ cleavage of the protected peptide prior to cyclization and subsequently a ‘strong’ deprotection. This may influence the yield of final peptide. In comparison, *N*-acyl peptides can be readily generated on resin and cleaved off from the resin by strong cleavage. Peptide can be then efficiently cyclized in a one pot buffer leading to high peptide yields.

The general applicability of the synthesis protocol was exemplified by the synthesis of a 50-residue long plant defensin. In principle, we have shown that a large, difficult to synthesize peptide can be ligated via two shorter fragments using the *N*-acylurea approach. Although we currently only have demonstrated the utility of this ligation approach in Cys containing peptides, other potential ligation sites such as Ala or Val broadens the applicability of this approach in the synthesis of peptides that do not contain Cys. For instance, desulfurization allows penicillamine to be converted into Val (Haase et al. 2008) and Cys into Ala (Wan and Danishefsky 2007). Hence, the current approach in combination with desulfurization can potentially be used to ligate peptides that lack Cys, given that they fulfill the requirement of an Ala or Val at the ligation site.

A user-friendly and time-efficient method of synthesis is a primary requirement to exploit the drug development aspects of cyclic peptides or to dissect their structure–activity relationships. As such the method presented in this study is timely, as many laboratories are becoming more inclined to use Fmoc SPPS rather than Boc SPPS and because of the increasing popularity of microwave assisted for peptide synthesis. In addition, the interest of using cyclic peptides as stable drug scaffolds is growing, and includes studies of naturally occurring cyclic peptides but also to the re-engineering of linear peptides by cyclization of the peptide backbone. In addition to cyclotides and SFTI-1, which both are currently scrutinized as scaffolds for drug discovery, current examples include the development of HIV inhibitors from retrocyclins, synthetic cyclic peptides derived from the primate θ defensin genes (Penberthy et al. 2011), and the cyclization of the conotoxin Vc1.1, which is now undergoing preclinical development for the treatment of neuropathic pain (Carstens et al. 2011). We anticipate that the protocol described in the current paper may advance the use of cyclic peptides for drug development, but also for a fragment based synthesis approach of larger proteins, exploiting the power of microwave assisted peptide synthesis.

## Conclusion

With the rising interest in cyclization as a tool to improve stability of peptides, the chemical synthesis of proteins, and the increasing use of microwave assisted peptide synthesis, there is a need for the continued development of new methods. The current work expands the applications of the C-terminal *N*-acylurea approach for NCL reported by (Blanco-Canosa and Dawson 2008). By including high-yielding and rapid microwave Fmoc SPPS in the protocol, the door is wide open for the efficient ligation of cyclic peptides or larger proteins by making ends meet.

## Electronic Supplementary Material

Below is the link to the electronic supplementary material.

Supplementary material 1 (DOC 23 kb)

Supplementary material 2 (DOC 745 kb)
