# Supplementary material for: Making Ends Meet: Microwave-Accelerated Synthesis of Cyclic and Disulfide Rich Proteins Via In Situ Thioesterification and Native Chemical Ligation
Source: Int J Pept Res Ther. 2012 Oct 14;19(1):43–54. doi: 10.1007/s10989-012-9331-y (PMC3597280; doi:10.1007/s10989-012-9331-y)
Supplement: Supplementary file 1 — Supplementary material 1 (DOC 23 kb) [file 10989_2012_9331_MOESM1_ESM.doc]

**Supplementary Information**

**Making ends meet: Microwave-accelerated synthesis of cyclic and disulfide rich proteins via *in situ* thioesterification and native chemical ligation**

Sunithi Gunasekera^1^, Teshome L. Aboye^1^, Walid A. Madian^1,2^, Hesham R. El-Seedi^1,2^, Ulf Göransson^1^*

^1^Division of Pharmacognosy, Department of Medicinal Chemistry, Uppsala University, Biomedical Centre, Box 574, SE-751 23 Uppsala, Sweden; ^2^Department of Chemistry, Faculty of Science, El-Menoufia University, 32512, Shebin El-Kom, Egypt.

*To whom correspondence should be addressed. Tel: +46 184715031.

Fax: +46 18509101. E-mail: [ulf.göransson@fkog.uu.se](mailto:ulf.gˆransson@fkog.uu.se)
