# Supplementary material for: Making Ends Meet: Microwave-Accelerated Synthesis of Cyclic and Disulfide Rich Proteins Via In Situ Thioesterification and Native Chemical Ligation
Source: Int J Pept Res Ther. 2012 Oct 14;19(1):43–54. doi: 10.1007/s10989-012-9331-y (PMC3597280; doi:10.1007/s10989-012-9331-y)
Supplement: Supplementary file 2 — Supplementary material 2 (DOC 745 kb) [file 10989_2012_9331_MOESM2_ESM.doc]

**Supplementary Information**

**Making ends meet: Microwave-accelerated synthesis of cyclic and disulfide rich proteins via *in situ* thioesterification and native chemical ligation**

**Calculation of peptide-Dbz yield for kalata-Bz**

Resin substitution value = 0.19 mol/g

Synthesis scale = 0.1 mM

Starting resin weight = 0.526g

Peptide-Dbz molecular weight with protecting groups, including N-terminal Boc = 5740 g/mol

Expected amount of peptide-Dbz mass from a 0.1 mM synthesis = 0.574 g

Expected peptide-resin weight after synthesis = 1.1g

Deprotected peptide-Dbz mass = 3050 g/mol

Expected amount of deprotected Dbz peptide from a 0.1 mM scale ynthesis = 0.305g

Amount of expected peptide from 50 mg of cleavage = 0.305/1.1 X 0.05 = 13.8 mg

Amount of actual peptide obtained from 50 mg of cleavage = 8.7g

% yield = 63%

**Calculation of peptide-Nbz yield for kalata B1**

Resin substitution value = 0.19 mol/g

Synthesis scale = 0.1 mM

Starting resin weight = 0.526g

Peptide-Nbz molecular weight with protecting groups, including N-terminal Boc = 5766 g/mol

Expected amount of peptide-Nbz mass from a 0.1 mM synthesis,= 0.577g

Expected peptide-resin weight after synthesis = 1.1 g

Deprotected peptide-Nbz mass = 3076 g/mol

Expected amount of deprotected Nbz peptide from 1.1 g of resin cleavage = 0.3076 mg

Amount of expected peptide from 100 mg of cleavage = 0.3076 /1.1 X 0.10 = 27.9 mg

Actual peptide from 100 mg of cleavage = 17.4 mg

% yield of Nbz peptide = 62.4%

**Kalata B1 Yield**

Native kalata B1 molecular weight = 2892 g/mol

Expected amount of native peptide from 0.1 mM synthesis/1.1 g of resin-Nbz peptide conversion = 0.289 g

Expected amount of native peptide from 0.1 g of resin-Nbz peptide conversion = 26.2 g

Actual amount of cyclized peptide from 0.1 g of resin-Nbz peptide conversion = 4.2 mg

kalata B1 yield = 16%

**Calculation of peptide-Dbz yield for SFTI-1(e)**

Resin substitution value = 0.19 mol/g

Synthesis scale = 0.1 mM

Starting resin weight = 0.526g

Peptide-Dbz molecular weight with protecting groups, including N-terminal Boc = 2920 g/mol

Expected amount of peptide-Dbz mass from a 0.1 mM synthesis = 0.292 g

Expected peptide-resin weight after synthesis = 0.818g

Deprotected peptide-Dbz mass = 1667 g/mol

Expected amount of deprotected Dbz peptide after synthesis = 0.1667g

Amount of expected peptide from 50 mg of cleavage = 0.1667/0.818 X 0.05 = 10.2 mg

Actual peptide from 50 mg of cleavage = 8.3

% yield = 81.3 %

**Calculation of peptide-Dbz yield for SFTI-1(f)**

Resin substitution value = 0.19 mol/g

Synthesis scale = 0.1 mM

Starting resin weight = 0.526g

Peptide-Dbz molecular weight with protecting groups, including N-terminal Boc = 2770 g/mol

Expected amount of peptide-Dbz mass from a 0.1 mM synthesis = 0.277 g

Expected peptide-resin weight after synthesis = 0.803g

Deprotected peptide-Dbz mass = 1667 g/mol

Expected amount of deprotected Dbz peptide after synthesis = 0.1667g

Amount of expected peptide from 50 mg of cleavage = 0.1667/0.803 X 0.05 = 10.4 mg

Actual peptide from 50 mg of cleavage = 8.1

% yield = 77.8 %

**Calculation of peptide-Nbz yield for SFTI-1**

Resin substitution value = 0.19 mol/g

Synthesis scale = 0.1 mM

Starting resin weight = 0.526g

Peptide-Nbz molecular weight with protecting groups, including N-terminal Boc = 2946 g/mol

Expected amount of peptide-Nbz mass from a 0.1 mM synthesis,= 0.2946 g

Expected peptide-resin weight after synthesis = 0.8206 g

Deprotected peptide-Nbz mass = 1693 g/mol

Expected amount of deprotected Nbz peptide from 0.8206 g of resin cleavage = 0.1693 mg

Amount of expected peptide from 100 mg of cleavage = 0.1693 /0.8206 X 0.10 = 20.6 mg

Actual peptide from 100 mg of cleavage = 18.3 mg

% yield of Nbz peptide = 88.8 %

**SFTI-1 Yield**

Native SFTI-1 molecular weight = 1513 g/mol

Expected amount of native peptide from 0.1 mM synthesis/0.8206 g of resin-Nbz peptide conversion = 0.1513 g

Expected amount of native peptide from 0.1 g of resin-Nbz peptide conversion = 18.4 g

Actual amount of peptide cyclized peptide from 0.1 g of resin-Nbz peptide conversion = 9.1 mg

Kalata B1 yield = 49.5 %

**Calculation of peptide-Dbz yield for Defensin peptide 1**

Resin substitution value = 0.4 mol/g

Synthesis scale = 0.25 mM

Starting resin weight = 0.625 g

Peptide-Dbz molecular weight with protecting groups, including N-terminal Boc = 5965.4 g/mol

Expected amount of peptide-Dbz mass from a 0.25 mM synthesis = 1.49135 g

Expected peptide-resin weight after synthesis = 2.11635

Deprotected peptide-Dbz mass = 3842 g/mol

Expected amount of deprotected Dbz peptide after synthesis = 0.3842g

Amount of expected peptide from 50 mg of cleavage = 0.3842/2.11635 X 0.05 = 9.08 mg

Actual peptide from 50 mg of cleavage = 5.1 mg

% yield = 56.1%

**Calculation of peptide-Nbz yield for Defensin peptide 1**

Resin substitution value = 0.4 mol/g

Synthesis scale = 0.25 mM

Starting resin weight = 0.625g

Peptide-Nbz molecular weight with protecting groups, including N-terminal Boc = 5991.4 g/mol

Expected amount of peptide-Nbz mass from a 0.25 mM synthesis,= 1.49785g

Expected peptide-resin weight after synthesis = 2.12285 g

Deprotected peptide-Nbz mass = 3076 g/mol

Expected amount of deprotected Nbz peptide from 2.1185 g of resin cleavage = 0.3076 mg

Amount of expected peptide from 45 mg of cleavage = 0.3076 /2.12285 X 0.045 = 6.5 mg

Actual peptide from 45 mg of cleavage = 3.5 mg

% yield of Nbz peptide = 53.8%


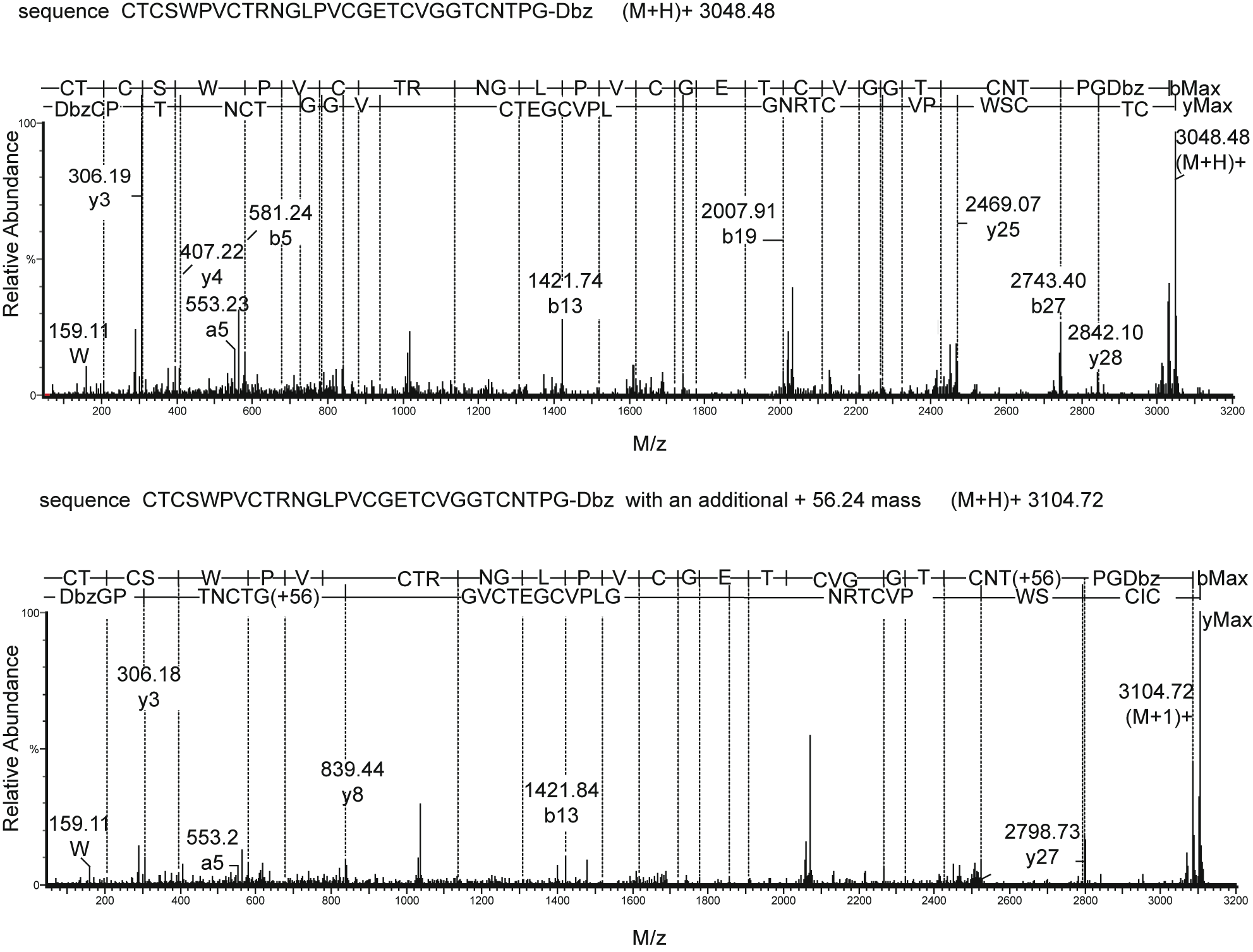


Fig.1 MS^2^ analysis of kalata B1. MS^2^ analysis of the parent peak is in the upper panel and the by-product in the lower panel.


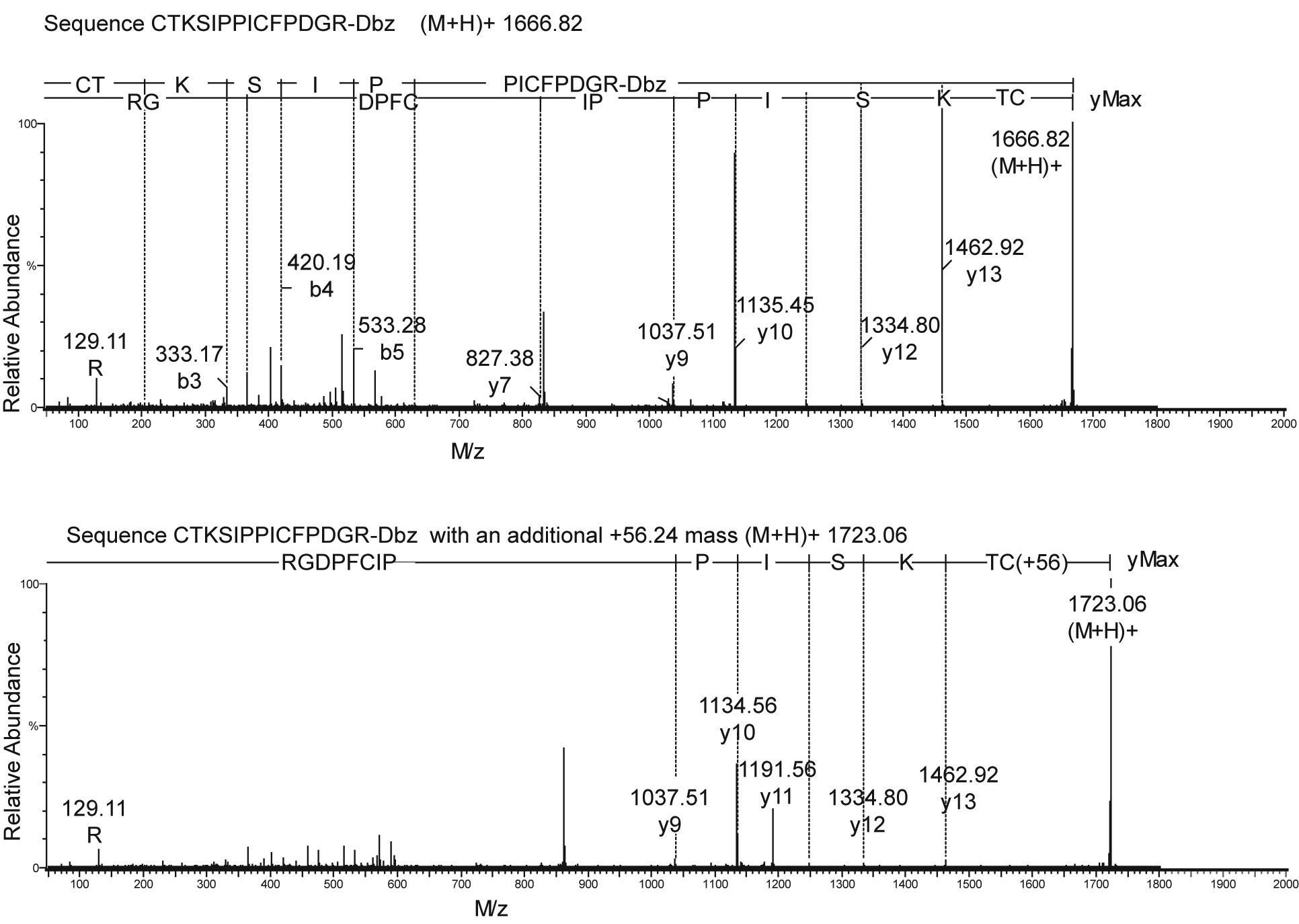


Fig.2 The MS^2^ analysis of SFTI-1(e). The upper panel shows the MS2 analysis of the native peak & the lower panel the by-product


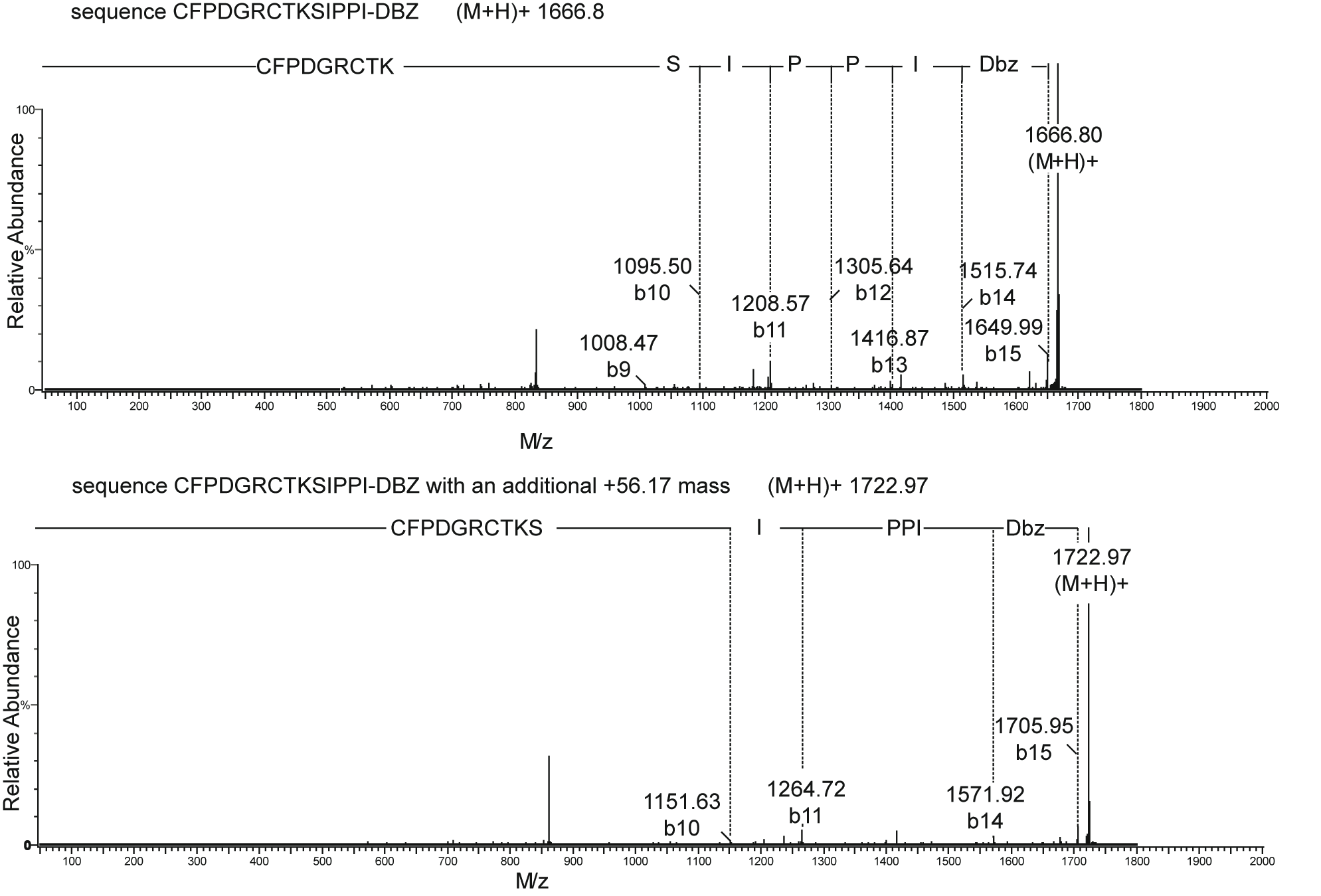


Fig. 3 MS^2^ analysis of SFTI-1(f). The upper panel indicates the MS^2^ analysis of the native peptide & the lower panel the by-product


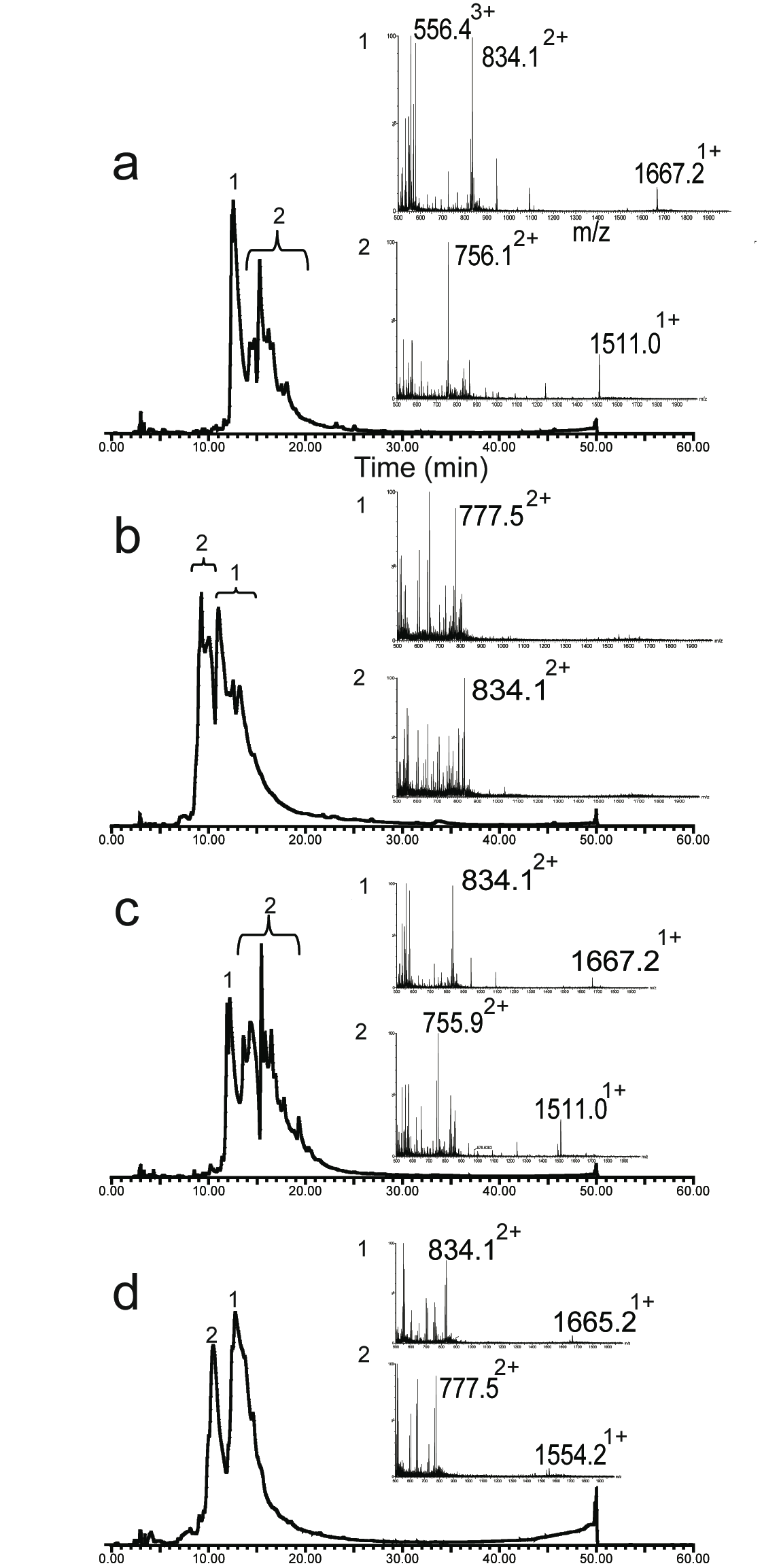


Fig. 4 RP-HPLC and MS analyses of SFTI-1synthesis trials. The different synthesis conditions used for the SFTI-1 variants a-d are highlighted in Table 1. Peak ‘1’ indicates Native SFTI-1 with (M+H)^+^ of 1667.2. The parentheses highlight the peaks containing deletion products in each synthesis trial. Peak ‘2’ represents the main deletion product for each synthesis trial; An Arg deletion is indicated by (M+H)^+^ of 1511.0; Ile deletion is indicated by (M+H)+ of 1554.2

Table 1: Synthesis conditions used in the synthesis of SFTI-1

| SFTI-1 trial | First residue | Reagents | Coupling duration and temperature |
| --- | --- | --- | --- |
| a  b  c  d | Arg  Ile  Arg  Ile | aa (4 eq), HBTU (4 eq), DIPEA (6 eq)  aa (4 eq), HBTU (4 eq), DIPEA (6 eq)  aa (7.5 eq), HBTU (7.5 eq), DIPEA (11.5 eq)  aa (7.5 eq), HBTU (7.5 eq), DIPEA (11.5 eq) | 1 hr x 2 (25ºC)  1 hr x 2 (25ºC)  1 hr x 2 (25ºC), 5 min x 1 (75ºC)  1 hr x 2 (25ºC) |
